# Experimental early‐life febrile seizures cause a sustained increase in excitatory neurotransmission in newborn dentate granule cells

**DOI:** 10.1002/brb3.2505

**Published:** 2022-02-22

**Authors:** Govert Hoogland, Marjolein Raijmakers, Elke Clynen, Bert Brône, Jean‐Michel Rigo, Ann Swijsen

**Affiliations:** ^1^ Department of Neurosurgery School for Mental Health and Neuroscience Maastricht University Medical Center Maastricht The Netherlands; ^2^ Neurophysiology Lab Biomedical Research Institute BIOMED Hasselt University Hasselt Belgium

**Keywords:** dentate granule cells, epileptogenesis, febrile seizures, hyperexcitability, neurogenesis

## Abstract

Prolonged febrile seizures (FS) are a risk factor for the development of hippocampal‐associated temporal lobe epilepsy. The dentate gyrus is the major gateway to the hippocampal network and one of the sites in the brain where neurogenesis continues postnatally. Previously, we found that experimental FS increase the survival rate and structural integration of newborn dentate granule cells (DGCs). In addition, mature post‐FS born DGCs express an altered receptor panel. Here, we aimed to study if these molecular and structural changes are accompanied by an altered cellular functioning. Experimental FS were induced by hyperthermia in 10‐days‐old Sprague‐Dawley rats. Proliferating progenitor cells were labeled the next day by injecting green fluorescent protein expressing retroviral particles bilaterally in the dentate gyri. Eight weeks later, spontaneous excitatory and inhibitory postsynaptic events (sEPSCs and sIPSCs, respectively) were recorded from labeled DGCs using the whole‐cell patch‐clamp technique. Experimental FS resulted in a robust decrease of the inter event interval (*p* < .0001) and a small decrease of the amplitude of sEPSCs (*p* < .001). Collectively the spontaneous excitatory charge transfer increased (*p* < .01). Experimental FS also slightly increased the frequency of sIPSCs (*p* < .05), while the amplitude of these events decreased strongly (*p* < .0001). The net inhibitory charge transfer remained unchanged. Experimental, early‐life FS have a long‐term effect on post‐FS born DGCs, as they display an increased spontaneous excitatory input when matured. It remains to be established if this presents a mechanism for FS‐induced epileptogenesis.

## Graphical abstract

Experimental, early‐life febrile seizures (FS) have a long‐term effect on post‐FS born dentate granule cells (DGCs). Here we show that, after inducing FS in 10‐days‐old rats and labeling the DGCs, these cells display an increased spontaneous excitatory input using whole‐cell patch‐clamp when matured.

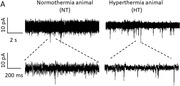

## 1 INTRODUCTION

Febrile seizures (FS) are fever‐induced seizures in children till the age of years and are considered a risk factor for contracting temporal lobe epilepsy (TLE) (Berg & Shinnar, 1996; Hauser, 1994). The suggestion that FS cause or signify hippocampal injury or aggravate a preexisting hippocampal lesion, thereby contributing to the development of TLE, comes from clinical retrospective cohort and case reports, and experimental studies (Abou‐Khalil et al., 1993; French et al., 1993; Hamati‐Haddad & Abou‐Khalil, 1998). One hypothesis is that FS initiate the development of a hyperexcitable hippocampal circuitry.

Within the hippocampal formation, the dentate gyrus (DG) is a major source of input that functions as a gatekeeper. Here the excitable input is controlled by inhibitory postsynaptic currents (sIPSCs) in dentate granule cells (DGCs). Experimental FS, a known risk factor for TLE, have shown to increase the amplitude of sIPSCs. However, also a decrease in frequency was found resulting in an unaltered net charge transfer (Swijsen, Avila, et al., 2012). On the short term, the glutamatergic transmission and LTP are not affected in the DG by hyperthermia treatment (van Campen et al., 2018). Another feature of the DG is that it is one of three regions where neurogenesis continues during adulthood (Altman & Das, 1965; Danzer, 2019; Eriksson et al., 1998). Neurogenic activity is especially high at the ages when FS occur. Previously we have shown that experimental FS increase the number of surviving post‐FS born DGCs and that these matured cells show a higher degree of dendritic complexity (Raijmakers et al., 2016). This was also confirmed in other experimental FS models (Gibbs et al., 2011; van Campen et al., 2018). Also, animal models for TLE are characterized by a seizure‐induced altered morphology of DGCs, increase in mitotic activity, accelerated maturation and integration (Jessberger et al., 2007; Parent et al., 1997; Singh et al., 2013). Though, five days after seizure induction DGCs already respond to glutamatergic perforant path stimulation (Overstreet‐Wadiche et al., 2006), these cells receive increased GABAergic synaptic input once they are fully integrated (Jakubs et al., 2006). Since GABA has an initial depolarizing effect on newborn cells (Ben‐Ari, 2002), this may further enhance hyperexcitability and thereby promote maturation and synaptic integration of these cells (Ge et al., 2006).

Experimental FS can induce ectopic expression of DGCs, that display increased excitatory GABAergic signaling (Koyama et al., 2012). As most post‐FS born DGCs migrate normotopically, we aimed to analyze whether these normotopic newborn cells also display an altered functioning. To this end, we modeled FS by exposing 10‐days‐old rats to a stream of heated air. Proliferating progenitor cells were labelled the next day by an intrahippocampal injection with retroviral particles expressing enhanced green fluorescent protein (eGFP). Eight weeks later, spontaneous excitatory and inhibitory postsynaptic currents (sEPSCs and sIPSCs, respectively) were measured using whole‐cell patch‐clamp recordings of eGFP‐positive newborn DGCs.

## 2 METHODS

### 2.1 Induction of experimental FS

A group of 6 to 10 male Sprague‐Dawley rats (Harlan, Horst, The Netherlands, RRID:MGI:5651135), culled from different litters, was housed together with a foster dam. Housing was temperature‐controlled (21±1°C), with a 12 h light/dark cycle and ad libitum access to food and water. All animal procedures were approved by the animal ethics committee of Hasselt University (ethical matrix 2012/42; 2013/56).

FS were induced as described earlier (Baram et al., 1997; Lemmens et al., 2005; Swijsen, Nelissen et al., 2012). Briefly, on postnatal day (P)10, pups were placed in a perspex cylinder and exposed to a stream of regulated heated air (Baram et al., 1997; Lemmens et al., 2005; Raijmakers et al., 2016; Swijsen, Avila, et al., 2012; Swijsen, Brone et al., 2012; Swijsen, Nelissen, et al., 2012). During this treatment, body temperature was measured rectally every 2.5 min and maintained for 35 min between 39.5 and 42.5°C. The occurrence of seizures, characterized by clonic contractions of fore‐ and hind limbs while lying on side or back, was monitored by two observers independently. Hyperthermia‐induced seizures typically lasted 8–9 min (Jansen et al., 2008; Lemmens et al., 2005). Animals not displaying behavioral seizures were excluded from the study. After hyperthermia treatment (HT), pups were cooled down to pre‐exposure body temperature by rubbing them with room temperature water‐soaked paper wipes and then returned to their nest. Control pups underwent the same procedure, with the exception that the stream of heated air was adjusted to keep them normotherm (NT), that is, to the body temperature that was measured at the start of the experiment (∼35°C). On P22, the dam was removed from the nest and from P36 onward animals were housed individually.

### 2.2 Labelling of newborn DG cells

Three plasmids, that is, a packaging plasmid encoding viral proteins (gag/pol) driven by a cytomegalovirus (CMV) promotor, an envelope plasmid encoding glycoprotein G of vesicular stomatitis virus (VSVG) and a transfer plasmid encoding eGFP driven by a CAG promotor (kindly donated by prof. H. van Praag, Laboratory of Neurosciences, Biomedical Research Center NIA, Baltimore, USA; van Praag et al., 2002), were transfected (at 37°C) with a mixture of lipofectamine 2000 (Invitrogen, Belgium) and Opti‐MEM (Invitrogen) in human embryonic kidney 293T cells to produce replication‐deficient enhanced GFP (eGFP)‐expressing retroviral particles based on the Moloney murine leukemia virus (MuLV) (Raijmakers et al., 2016). These eGFP‐expressing retroviral particles only label proliferating cells as MuLV based retroviral vectors need passage through mitosis to integrate into cells (Lewis & Emerman, 1994; van Praag et al., 2002). Supernatant containing eGFP‐expressing retroviral particles was collected after 72 h, filtered through a 0.22 μm filter and concentrated by two centrifugations of 19,400 relative centrifugal force (rcf) (2 h at 4°C). This typically resulted in a titer of 5.10^11^ viral particles/ml (determined by QuickTiterTM Retrovirus Quantitation Kit, BioConnect, the Netherlands; data not shown). The final pellet was resuspended in phosphate‐buffered saline and stored at −80°C.

On P11, pups were anesthetized by a subcutaneous injection containing 37.5 μg/kg dexmedetomidine hydrochloride (Dexdomitor^®^, Orion Corporation, Finland) and 75 μg/kg buprenorphine hydrochloride (Temgesic^®^, Reckitt Benckiser Healthcare, United Kingdom), followed 10 min later by an intraperitoneal injection of 22.5 mg/kg ketamine hydrochloride (Ketalar^®^, Pfizer, Ireland). Pups were placed on a temperature‐controlled heating pad in a stereotactic frame with a neonatal rat adaptor (Stoelting, Illinois, USA). After incision of the skin, two drops of 2% lidocaine hydrochloride (Xylocaine^®^, Recipharm Monts, France) were applied on the periost. Holes were drilled bilaterally in the skull (anteroposterior, −3.0 mm; mediolateral, 2.4 mm; both coordinates relative to lambda). Concentrated eGFP‐expressing particles were bilaterally injected in the dentate gyri at 0.25 μl/min using a 34‐gauge beveled needle (World Precision Instruments, United Kingdom), that is, each dentate gyrus received 1 μl at dorsoventral coordinate −3.1 mm and 1 μl at dorsoventral coordinate −2.6 mm. The skin was closed using 3/0 Vicryl (Johnson & Johnson, Belgium). Upon recovery from anesthesia, the animal was returned to its nest. One day post‐surgery, animals received a subcutaneous injection of 1.5 mg/kg meloxicam (Metacam^®^, Labiana Life Sciences S.A., Spain) for pain relief.

### 2.3 Tissue preparation

On P66, animals used for sEPSC measurements were sacrificed by an intraperitoneal injection with 100 mg sodium pentobarbital (sodium Nembutal^®^, Ceva Santé Animale, France) per kg bodyweight. Animals used for sIPSC measurements first received a subcutaneous injection with 37.5 μg/kg dexmedetomidine hydrochloride (Dexdomitor^®^, Orion Corporation, Finland) and 10 min later an intraperitoneal injection with 100 mg/kg ketamine hydrochloride (Ketalar^®^, Pfizer, Ireland). Subsequently, animals were transcardially perfused with ice‐cold bubbled (95%O2/5%CO2) NMDG‐HEPES solution, containing in mM: 92 NMDG, 2.5 KCl, 1.25 NaH_2_PO_4_, 30 NaHCO_3_, 20 HEPES, 25 Glucose, 5 Na‐ascorbate, 2 thiourea, 3 Na‐pyruvate, 10 MgSO_4_.H_2_O and 0.5 CaCl_2_.2H_2_O (pH 7.3, ∼310 mOsm). Next, brains were removed quickly, submerged in ice‐cold bubbled NMDG‐HEPES solution and 300 μm thick coronal slices were cut using a Leica VT1200 S microtome. Slices were recovered for 12 min in bubbled NMDG‐HEPES solution and then transferred to room temperature (RT) oxygenated HEPES recovery solution containing in mM: 92 NaCl, 2.5 KCl, 1.25 NaH_2_PO_4_, 30 NaHCO_3_, 20 HEPES, 25 Glucose, 5 Na‐ascorbate, 2 thiourea, 3 Na‐pyruvate, 2 MgSO_4_.H_2_O and 2 CaCl_2_.2H_2_O (pH 7.3, ∼310 mOsm) in which they were kept until electrophysiological measurements.

### 2.4 Whole‐cell patch‐clamp recordings

For measurements, individual slices were placed in a recording chamber and slices were continuously superfused with oxygenated aCSF at room temperature, containing in mM: 124 NaCl, 2CaCl_2_.2H_2_O, 3 KCl, 10 Glucose, 26 NaHCO_3_, 1.25 NaH_2_PO_4_.2H_2_O and 1 MgCl_2_. The granular layer of the DG was visualized using an upright Nikon microscope (Eclipse FN1; Nikon BeLux, Brussels, Belgium) with a 40× water‐immersion objective and infrared‐sensitive CCD video camera (KP‐M2RP, Hitachi Kokusai Electric Europe, Erkrath, Germany). Normotopic eGFP‐positive DG cells were selected for individual measurements. Recording electrodes were pulled from borosilicate glass using a P‐97 Flaming‐Brown horizontal puller (Sutter Instruments, Novato, CA, USA). Electrodes had a resistance of 4–7 MΩ and were fire polished using a microforge (Narishige). Cells were clamped in the whole‐cell configuration at a holding potential of −60 mV. For sEPSC measurements, electrodes were filled with buffer containing in mM: 130 KCl, 5 NaCl, 1 CaCl_2_.2H_2_O, 1 MgCl_2_, 10 HEPES, 10 EGTA, 2 NaATP and 0.5 NaGTP (pH 7.3, ∼270 mOsm) and aCSF containing 20 μM GABAzine was applied via a fast application perfusion system (SF‐77B, Warner Instruments, Holliston, MA, USA) to eliminate GABA_A_ receptor mediated currents. For sIPSC measurements, electrodes were filled with a buffer containing in mM: 137 CsCl, 5 MgCl_2_, 10 HEPES, 10 EGTA, 1 CaCl_2_, 4 Na‐ATP, 0.4 Na‐GTP and 5 QX‐314 (lidocaine N‐ethyl bromide) (pH 7.3, ∼290 mOsm) and aCSF containing 50 μM DL‐2‐amino‐5‐phosphonopentanoic acid (DL‐APV) and 20 μM 6‐cyano‐7‐nitroquinoxaline‐2,3‐dione (CNQX) was applied via a fast application perfusion system to block both N‐methyl‐D‐aspartate (NMDA) and α‐amino‐3‐hydroxy‐5‐methyl‐4‐isoxazolepropionic acid (AMPA)/kainate receptor‐mediated currents. Currents were amplified by an Axopatch 200B Patch‐Clamp amplifier (http://www.moleculardevices.com, RRID:SCR_018866). Recordings of spontaneous activity were collected over 120 s and low‐pass filtered at 1–5 kHz. Data was collected and digitized (20 kHz) with PClamp 10.0 (Molecular Devices) and analyzed off‐line.

### 2.5 Electrophysiological data analysis

A PClamp 10.0 software package (http://www.moleculardevices.com, RRID:SCR_011323) and Mini Analysis software (http://www.synaptosoft.com, RRID:SCR_002184) with a threshold set at five times the root mean square noise were used for automatic detection of spontaneous currents. All events were checked manually and artifacts were excluded from analyses. These data were then used to calculate the interevent interval, amplitudes and 10–90% rise time of the events. The charge transferred by each individual event was calculated by determining the area under the event waveform. The event charge transfer per second was determined by multiplying the mean number of events per second by the mean charge transfer per event. For non‐stationary noise analysis an ensemble of events was aligned by the point of 50% rise time to construct an averaged event. Only cells with a precise event alignment were included. Potential interference due to overlaying events was controlled by including only events that were more than 200 ms distant from another event or artifact. The equation *σ^2^ = iI – I^2^ / N + b*1 (*σ^2^* = variance, *i* = unitary current, *I* = mean current, *N* is the estimated number of channels that are activated at the peak of the mean current, and *b*1 = background variance) was used to calculate the estimated number of channels that were open at the peak of the mean current and the unitary current.

### 2.6 Statistical analysis

Statistical analyses were performed using GraphPad Prism 9 software (http://www.graphpad.com, RRID:SCR_002798). The inter event interval, amplitude and 10–90% rise time analysis were expressed as cumulative probability and tested by a two sample Kolmogorov‐Smirnov test. Charge transfer, number of open channels at peak amplitude and the unitary current were tested nonparametrically by a Mann–Whitney test, as data were not normally distributed, and expressed as median with interquartile range and whiskers (5–95% interval). A *p*‐value < .05 was considered as significant. The number of cells is represented by n.

## 3 RESULTS

**FIGURE 1:**
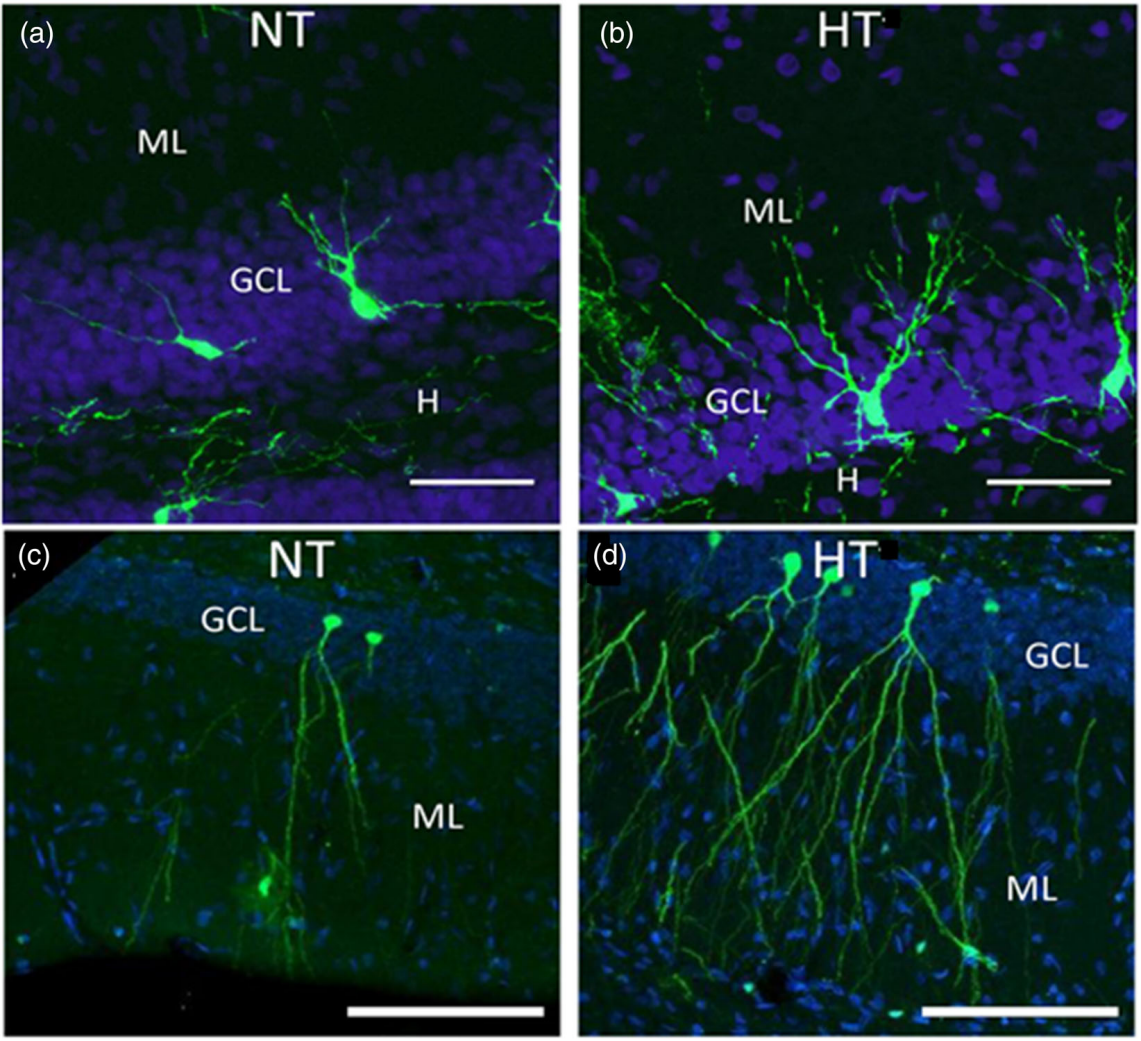
Photomicrographs of hippocampal eGFP‐labeled DGCs that were born after normothermia (NT) or hyperthermia (HT) treatment and matured for 1 week (a and b, respectively) or 8 weeks (c and d, respectively). After 1 week of differentiation, post‐FS born DGCs show longer dendrites and after 8 weeks of differentiation they show increased dendritic complexity, compared to DGCs in NT controls. Also, we previously observed that the number of mushroom spines is significantly increased at 8 weeks after HT (Raijmakers et al., 2016). Nuclei stained blue with DAPI. GCL, granular cell layer; H, hilus; ML, molecular layer; scale bar = 50 μm (adapted from Raijmakers et al. Epilepsia 2016;57(5):717‐726)

Previous work indicated that post‐FS born DGCs display structural modifications at a faster pace, compared to DGCs in control animals (Raijmakers et al., 2016). At one week after experimental FS induction, dendrites of post‐FS born cells only reached the border of the DG/molecular layer (Raijmakers et al., 2016) and cells were non‐responsive to synaptic input (data not shown). At eight weeks after FS, dendrites of these cells reached both the inner and outer molecular layer (Figure 1). In the present study, we assessed the synaptic connectivity of 8‐weeks‐old DGCs that were born 24 h after FS by recording their spontaneous postsynaptic currents.

### 3.1 Experimental FS cause a sustained increase in excitatory input to post‐FS born DGCs

**FIGURE 2:**
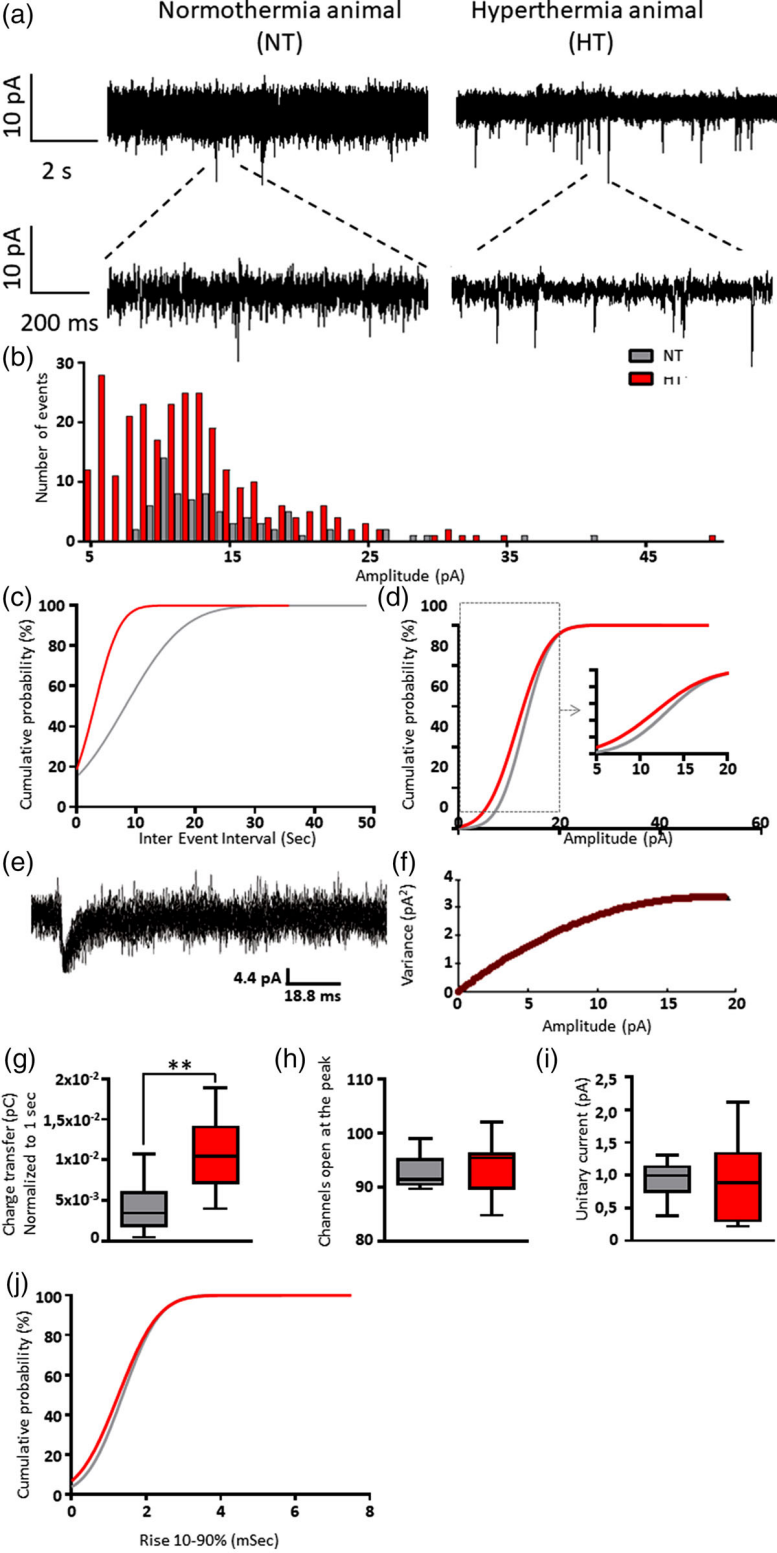
Electrophysiological properties of sEPSCs in DGCs. Ten‐days‐old rats were submitted to a 30 min normothermia (NT) or hyperthermia (HT) treatment. The next day, the animals received an intrahippocampal injection with eGFP‐expressing viral particles to label newborn DGCs. After 8 weeks, sEPSCs of labeled DGCs were recorded by a whole‐cell patch‐clamp configuration in the presence of 20 μM GABAzine. (a) Example traces from a DGC of a NT and of an HT animal. (b) Distributions of the amplitudes of sEPSCs recorded from 10 DGCs of NT controls and 12 DGCs of HT animals. (c) The inter‐event interval was smaller in DGCs from HT animals than in DGCs from controls (*p* < .0001), indicating an increase in frequency. (d) The amplitude of sEPSCs in HT animals was decreased compared to that in controls (*p* < .001). Example fit (e) and variance‐current plot (f) of a control animal. (g) The charge transfer of sEPSCs was increased in HT animals compared to that in controls (*p* < .01). (h) The estimated number of channels that were open at the peak of the mean current was similar in both animal groups. (i) Likewise, the unitary currents did not differ between groups. (j) Also, the 10–90% rise times of both groups were comparable. b, c, d, g, j: NT, 76 events, *n* = 10 from 6 animals; HT+, 278 events, *n* = 12 from 7 animals. h, i: NT, 61 events, *n* = 7 from 5 animals; HT+, 221 events, *n* = 9 from 6 animals

Excitatory synaptic activity was examined by recording sEPSCs in the presence of the GABA_A_ receptor blocker GABAzine to isolate excitatory glutamatergic currents. Figure 2a shows sEPSC recordings from 8‐weeks‐old DGCs that were born 24 h after induction of FS. Compared to NT‐treated controls, the distribution of sEPSC amplitudes in HT‐treated animals appeared more toward lower amplitudes (Figure 2b,d; *p* < .001). The number of sEPSCs was strongly increased in HT‐treated animals (Figure 2b), as also indicated by a reduced inter‐event interval (Figure 2c, *p* < .0001). To evaluate whether the increase in frequency and the decrease in amplitude resulted in a net change of excitatory neurotransmission, we multiplied the mean number of events per second by the mean charge transfer per event to obtain the total amount of charge transferred per second by sEPSCs. Collectively, the net charge transferred by sEPSCs was three times higher in DGCs from HT‐treated animals, than in DGCs from NT controls (Figure 2g, *p* < .01). Using non‐stationary noise analysis (Figure 2e,f), we next tested whether differences at the single channel level may explain the change in whole‐cell sEPSC amplitudes. The estimated number of channels (about 93) that were open at the peak of the averaged sEPSC event was similar in both animal groups (Figure 2h). Also, the estimated single channel or unitary current (about 1 pA) did not differ between groups (Figure 2i). Finally, analysis of the sEPSC kinetics showed that the 10–90% rise time in HT animals was comparable to that in NT controls (Figure 2j).

### 3.2 FS do not increase the net charge transfer of inhibitory currents of post‐FS born DGCs

**FIGURE 3:**
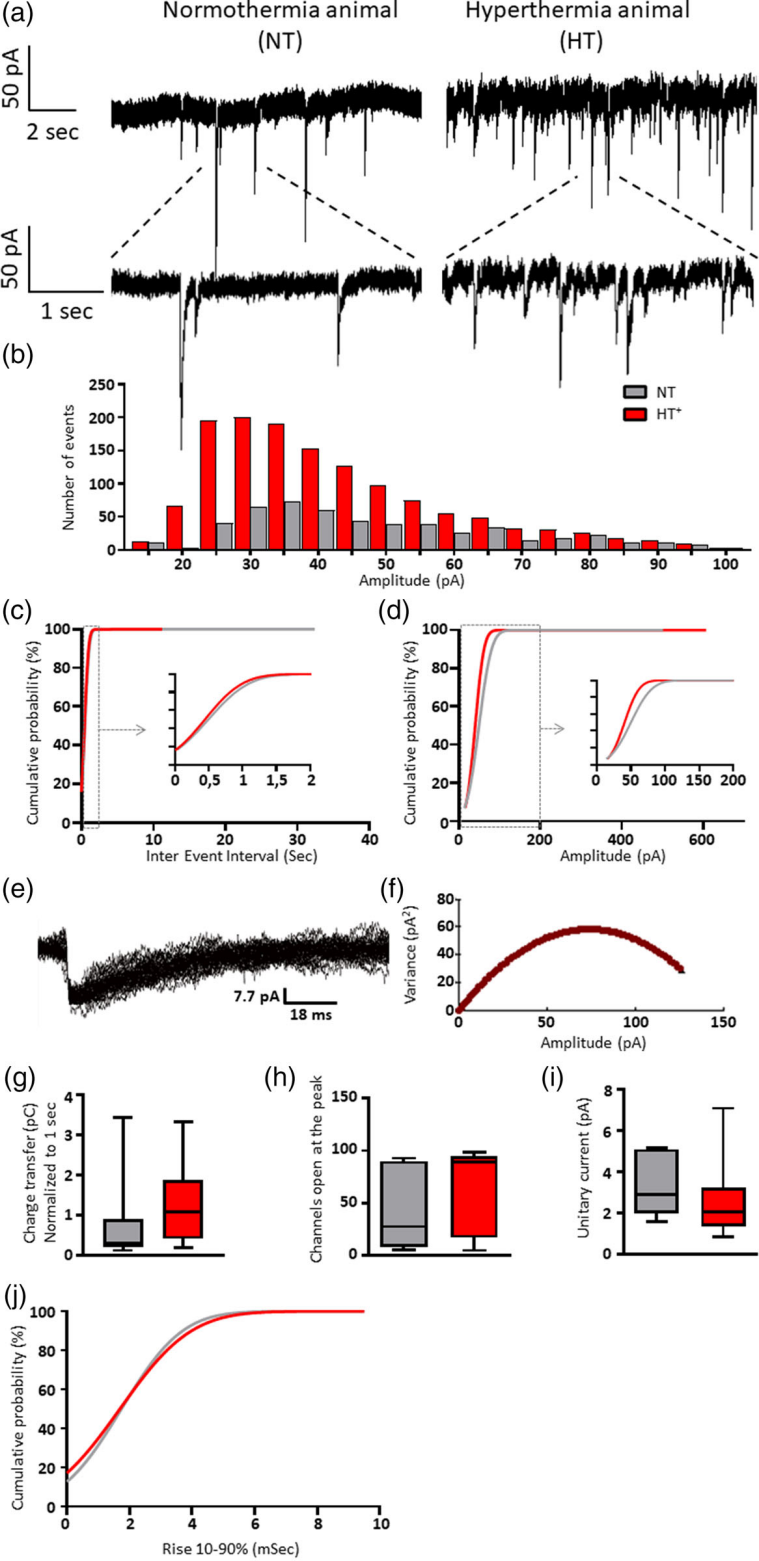
Electrophysiological properties of sIPSCs in DGCs. Ten‐days‐ old rats were submitted to a 35 min normothermia (NT) or hyperthermia (HT) treatment. The next day, the animals received an intrahippocampal injection with eGFP‐expressing viral particles to label newborn DG cells. After 8 weeks, sIPSCs of labelled DGCs were recorded by a whole‐cell patch‐clamp configuration with 5 mM QX‐314 (lidocaine N‐ethyl bromide) in the electrode, and 50 mM DL‐2‐amino‐5‐phosphonopentanoic acid (DL‐APV) and 20 μM 6‐cyano‐7‐nitroquinoxaline‐2,3‐dione (CNQX) superfused on the slice. (a) Example traces from a DGC of a NT and of an HT animal. (b) Distribution of the amplitudes of sIPSCs recorded from 9 DGCs of HT animals and 7 DGCs of NT controls (cut‐off at 100 pA). (c) The inter‐event interval was smaller in DGCs from HT animals than in DGCs from controls (*p* < .05), indicating an increase in frequency. (d) The amplitude of sIPSCs in HT animals was decreased compared to that in controls (*p* < .0001). Example fit (e) and variance‐current plot (f) of a control animal. (g) The charge transfer of sIPSCs in HT animals was similar to that in controls. (h) The estimated number of channels that were open at the peak of the mean current was similar in both animal groups. (i) Likewise, the unitary currents did not differ between groups. (j) The 10–90% rise times of HT animals was different from in controls (*p* < .01). b, c, d, g, j: NT, 573 events, *n* = 7 from 6 animals; HT+, 1403 events, *n* = 9 from 6 animals. h, i: NT, 136 events, *n* = 6 from 6 animals; HT+, 716 events, *n* = 8 from 5 animals

Next, we recorded sIPSCs to evaluate inhibitory synaptic transmission from post‐FS born DGCs. Spontaneous IPSC recordings were performed in the presence of the glutamate receptor blockers DL‐APV and CNQX in order to eliminate glutamatergic spontaneous events. Action potentials in the target neuron were blocked by adding the sodium channel blocker QX‐314 to the recording pipette. Figure 3a shows sIPSC recordings from 8‐weeks‐old DGCs that were born 24 h after induction of experimental FS. Compared to NT‐treated controls, the distribution of sIPSC amplitudes in HT‐treated animals strongly shifted toward lower amplitudes (Figures 3b,d; *p* < .0001). In addition, the number of sIPSCs was slightly increased in HT‐treated animals (Figure 3b, cut‐off at 100 pA), as also indicated by a reduced inter‐event interval (Figure 3c, *p* < .05). Collectively, the net charge transferred by sIPSCs in DGCs from HT‐treated animals was comparable to that in DGCs from NT controls (Figure 3g, fold change of 1.47). Also, the estimated number of channels that were open at the peak of the averaged sIPSC event was similar in both animal groups (Figure 3h), as well as the single channel or unitary current (Figure 3i, NT: 2,9 and HT 2,1 pA). Finally, analysis of sIPSC kinetics showed that the 10–90% rise times in HT‐treated animals were only slightly different from that in controls (*p* < .01; Figure 3j).

## 4 DISCUSSION

Neurogenesis in the DG has been hypothesized to play a role in the development of TLE. Several studies have reported an aberrant development and/or network integration of hippocampal cells that are born after seizures (Gibbs et al., 2011; Raijmakers et al., 2016; van Campen et al., 2018). Though the long‐term effect of FS on the behavior of the hippocampal network remains largely unknown, a significant proportion of animals develops limbic‐onset epilepsy (Dube et al., 2006). Previously, we observed that eight weeks after experimental FS the number of new DGCs was increased and that their dendrites developed faster and exhibited more mushroom spines and increased dendritic complexity (Figure 1; Lemmens et al., 2005; Raijmakers et al., 2016; Swijsen, Brone, et al., 2012). This suggests that experimental FS drive an increased production and accelerated synaptic integration of DGCs. In this study, we aimed to address the question if these morphological changes are accompanied by cell electrophysiological changes. By whole‐cell patch‐clamp, we measured spontaneous excitatory and inhibitory currents in post‐FS born, 8‐weeks‐old DGCs. Compared to 8‐weeks‐old DGCs from control animals, those from FS animals exhibited a similar inhibitory charge transfer and an increased excitatory input.

As these new DGCs are a minor part of the total number of DGCs it is not clear to what extend their increased excitability affects the dentate gyrus output and if this is a sign of epileptogenesis. By comparison, the pilocarpine model of TLE is also characterized by prolonged excitation of DGCs (Kobayashi & Buckmaster, 2003). In that model, it precedes the onset of spontaneous recurrent seizures by days to weeks suggesting that it may contribute, but is insufficient, to cause epilepsy. A cellular mechanism of excitatory neuronal activity is that it controls spine density (Swann et al., 2000). For instance, blocking the NMDA receptor results in an increased dendritic spine density while activation leads to a decreased density. Accordingly, in different TLE models, the excessive glutamate release during seizures result in a decreased spine density (Isokawa, 1998; Santos et al., 2011; Singh et al., 2013; Tejada et al., 2014). This may serve as a protective mechanism to counteract high DG activity during seizures. The higher dendritic complexity and increased number of mushroom spines on dendrites of post‐FS born, 8‐weeks‐old DGCs previously reported (Raijmakers et al., 2016), corroborates with the increased excitatory input reported here. Moreover, this increased excitatory input, reflected by an increase in sEPSC frequency, is accompanied by a decreased sEPSC amplitude resulting together in a higher net charge transfer by post‐FS born DGCs. Similar to the prior mentioned pilocarpine model, it is therefore tempting to speculate that these cellular changes are part of an epileptogenesis process. Alternatively, or additionally, the increased excitatory input to post‐FS born DGCs may also due to alterations of input circuits. Interestingly, we previously detected increased fiber density in the hippocampus two months after FS (Jansen et al., 2008). Also, Bender et al. (2003) revealed abnormal mossy fiber innervation of the granule cell and molecular layer three months after FS . In several animal seizure models, mossy fiber sprouting correlates with abnormal recurrent excitation of DGCs (Lynch & Sutula, 2000; Wuarin & Dudek, 2001). Yet, it remains speculative if this phenomenon of mossy fiber sprouting contributes to the observed altered excitatory input to post‐FS born DGCs.

In another FS model, Kwak et al. reported immunohistochemical findings of excitatory synaptogenesis in newly generated DGCs at seven weeks after FS. These morphological changes were accompanied by an elevated excitability ratio of DG paired‐pulse responses and preceded the onset of recurrent seizures at ten weeks after FS (Kwak et al., 2008). Also here, the contribution that these new DGCs may have had to alter the hippocampal network physiology remained unanswered. Interestingly, as soon as one week after pilocarpine‐induced seizures does perforant path stimulation result in glutamatergic input to new DGCs. By contrast, new DGCs in non‐seizure control animals do not show a functional integration at that time yet (Overstreet‐Wadiche et al., 2006). Additionally, seizures can induce aberrant migration of newborn DGCs, resulting in hilar dendrites and hilar DGCs with an increased excitatory input, which contributes to the development of epilepsy (Koyama et al., 2012; Ribak et al., 2000; Thind et al., 2008). The sensitivity of new DGCs to excitatory input shown here may thus contribute to hyperexcitability during the latent period following FS.

In addition to an increased excitatory state, we also found that the inhibitory charge transfer in new DGCs of FS animals was similar to that in controls. By patch‐clamp recordings one week after FS, we previously reported a decreased sIPSC frequency in DGCs (Swijsen, Avila, et al., 2012). Those recordings were made in unlabeled DGCs and therefore did not permit to distinguish pre‐FS, existing DGCs from post‐FS generated ones. The relatively small number of new DGCs however favors the assumption that these recordings mostly came from pre‐FS, existing DGCs. Moreover, our patch‐clamp recordings on 1‐week‐old post‐FS born DGCs revealed no inhibitory or excitatory synaptic activity in those cells (non‐published results). In contrast, the current observations were made in post‐FS generated DGCs, which show a slightly increased sIPSC frequency. As new DGCs are more plastic, this may be a mechanism to compensate the previously observed loss of inhibition in pre‐FS, existing DGCs. The iPSCs of post‐FS born DGCs also exhibited a decreased amplitude, possibly indicating a decreased number and/or altered receptor kinetics. The latter suggestion is further supported by the slight change in 10–90% rise time. Again, this characteristic may be specific to new DGCs as we previously reported an increased sIPSC amplitude in unlabeled DGCs (Swijsen, Avila, et al., 2012).

It is undisputed that brain plasticity, such as the degree of neurogenesis, decreases with age. For example, in adult rats, about 50% of newborn DGCs die within 4 weeks yet matured neurons are relatively stable. In contrast, in postnatal day 6 rats almost all newborn DGCs survive up to 8 weeks and about 20% of them die after reaching maturity at 5–6 months (Cahill et al., 2017). This suggests that a substantial number of DGCs that are born at infancy survive through young adulthood. It is known that mature DGCs regulate the recruitment of DGCs that are born in adulthood (Alvarez et al., 2016). Altering early‐life neurogenesis may therefore have a long‐lasting impact on the DGC network and presents a risk for pathogenesis as well as a target for modification of hippocampal (dys)functioning (Danzer, 2019).

In conclusion, these data show that experimental FS result in an increased excitatory and unaltered inhibitory input to mature, post‐FS born DGCs. The consequence of this cellular change to the overall hippocampal physiology and behavior remains to be established. Yet, this finding supports the hypothesis that neurogenesis can be a mechanism that facilitates FS‐induced epileptogenesis. Conclusive evidence for this suggestion may come from experiments in which post‐FS neurogenesis is ablated and followed up by detection of spontaneous recurrent seizures.

## CONFLICT OF INTEREST

The authors declare no conflict of interest.

### PEER REVIEW

The peer review history for this article is available at https://publons.com/publon/10.1002/brb3.2505

## Data Availability

The data that support the findings of this study are available from the corresponding author upon reasonable request.
